# Supplementary material for: Chromatin accessibility dynamics dictate renal tubular epithelial cell response to injury
Source: Nat Commun. 2022 Nov 28;13:7322. doi: 10.1038/s41467-022-34854-w (PMC9705299; doi:10.1038/s41467-022-34854-w)
Supplement: Supplementary file 1 — Supplementary information [file 41467_2022_34854_MOESM1_ESM.pdf]

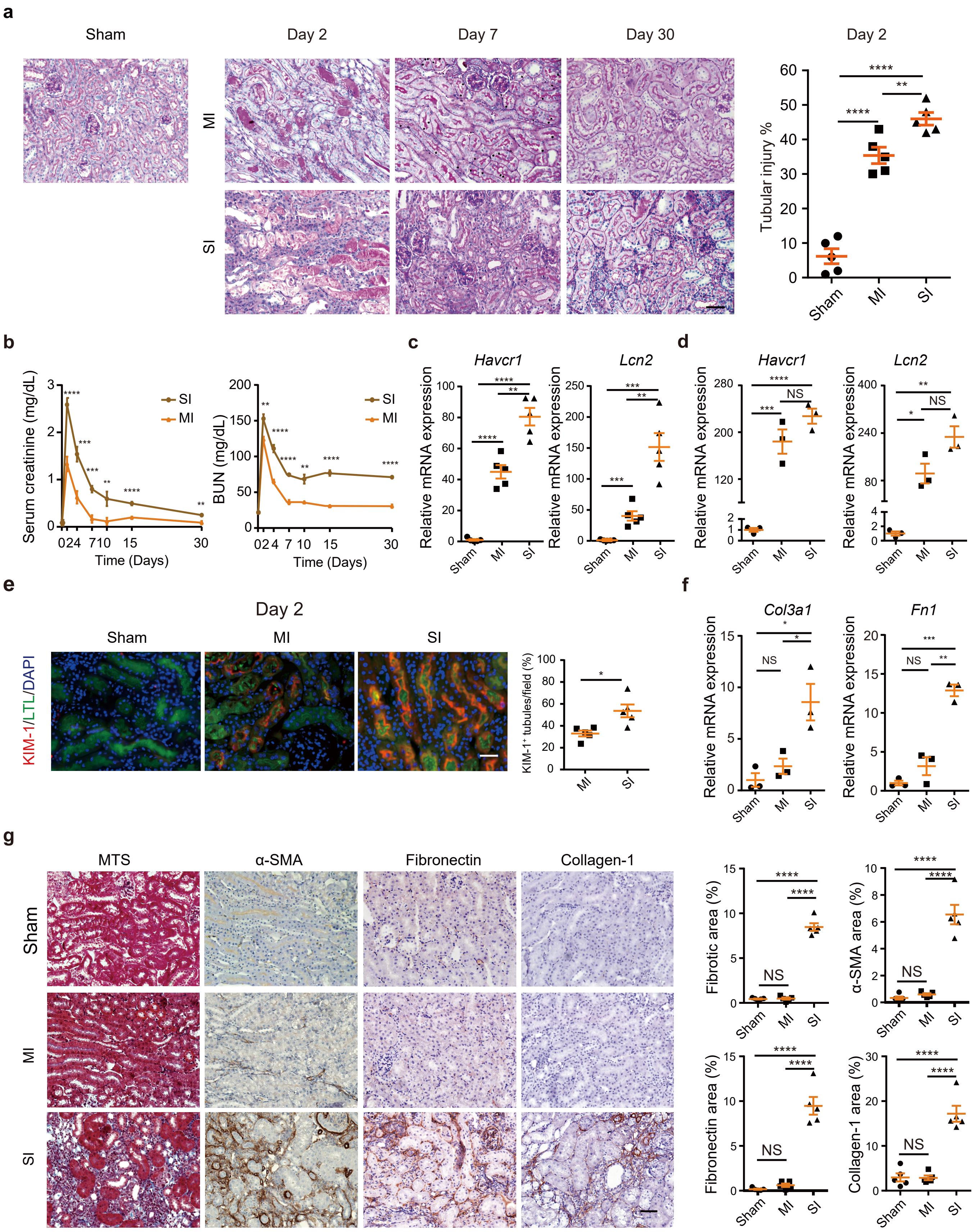
a
Sham
Day 2
Day 7
Day 30
Day 2
MI
SI
Tubular injury %
Sham
MI
SI
b
Serum creatinine (mg/dL)
Time (Days)
BUN (mg/dL)
Time (Days)
SI
MI
c
Havcr1
Lcn2
Relative mRNA expression
d
Havcr1
Lcn2
NS
e
Day 2
Sham
MI
SI
KIM-1/LTL/DAPI
KIM-1⁺ tubules/field (%)
f
Col3a1
Fn1
Relative mRNA expression
NS
g
MTS
α-SMA
Fibronectin
Collagen-1
Sham
MI
SI
Fibrotic area (%)
α-SMA area (%)
Fibronectin area (%)
Collagen-1 area (%)
NS

Supplementary Figure 1. Mouse models of mild injury and severe injury.
a Representative periodic acid-Schiff (PAS) staining of the kidneys (left). Scale bars, 50 µm. Tubular injury scores were analyzed (right). Data are expressed as means ± SEM. From left to right: ****$P < 0.0001$, ****$P < 0.0001$, **$P = 0.0076$, respectively, by two-tailed unpaired Student's *t*-test. **b** Serum creatinine (mg/dL) and BUN (mg/dL) in MI and SI mice. Data are expressed as means ± SEM. Statistically significant differences were determined by two-tailed unpaired Student's *t*-test. From left to right for Serum creatinine: ****$P < 0.0001$, ***$P = 0.0004$, ***$P = 0.0001$, **$P = 0.0063$, ****$P < 0.0001$, **$P = 0.0052$, respectively. From left to right for BUN: **$P = 0.0041$, ****$P < 0.0001$, ****$P < 0.0001$, **$P = 0.0012$, ****$P < 0.0001$, ****$P < 0.0001$, respectively. **c** qRT-PCR analysis of *Havcr1* and *Lcn2* expression in whole kidney. Data are expressed as means ± SEM. From left to right: ****$P < 0.0001$, ****$P < 0.0001$, **$P = 0.0011$, ***$P = 0.0002$, ***$P = 0.0009$, **$P = 0.0015$, by two-tailed unpaired Student's *t*-test. **d** qRT-PCR analysis of *Havcr1* and *Lcn2* expression in isolated TECs. Data are expressed as means ± SEM. From left to right: ****$P < 0.0001$, ***$P = 0.0009$, NS $P = 0.1497$, **$P = 0.0033$, *$P = 0.0364$, NS $P = 0.0655$, , by two-tailed unpaired Student's *t*-test. NS: not significant. $n = 3$ biologically independent samples. **e** Immunofluorescence staining of KIM-1 in MI and SI kidneys. Data are expressed as means ± SEM. Statistically significant differences were determined by two-tailed unpaired Student's *t*-test. *$P = 0.012$. Scale bar, 25 µm. **f** qRT-PCR analysis of *Col3a1* and *Fn1* expression in whole kidney. Data are expressed as means ± SEM. Statistically significant differences were determined by two-tailed unpaired Student's *t*-test. From left to right: *$P = 0.0162$, NS $P = 0.2543$, *$P = 0.0318$, ***$P = 0.0001$, NS $P = 0.1454$, **$P < 0.0021$, respectively. $n = 3$ biologically independent samples. **g** Representative images of Masson's trichrome (MTS) staining, immunohistochemistry staining of α-SMA, fibronectin, and collagen-1 in MI and SI kidneys. Scale bar, 50 µm. Statistically significant differences were determined by two-tailed unpaired Student's *t*-test. ****$P < 0.0001$. $n = 5$ biologically independent samples (a-**c**, **e**, and **g**). Source data are provided as a Source Data file.

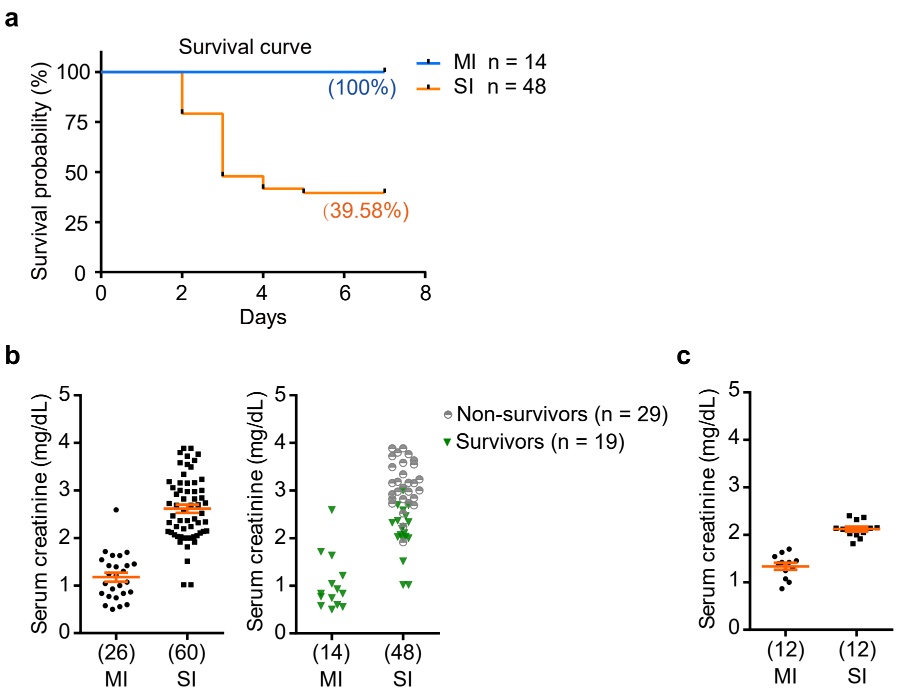

Supplementary Figure 2.
a Kaplan–Meier survival curves for mouse models of mild injury and severe injury. **b** Serum creatinine concentrations in MI and SI mice at day 2 (left). Non-survivors and survivors are labeled (right). **c** Serum creatinine concentrations in mice for ATAC-seq and RNA-seq. Data are expressed as means ± SEM (**b** and **c**). Source data are provided as a Source Data file.

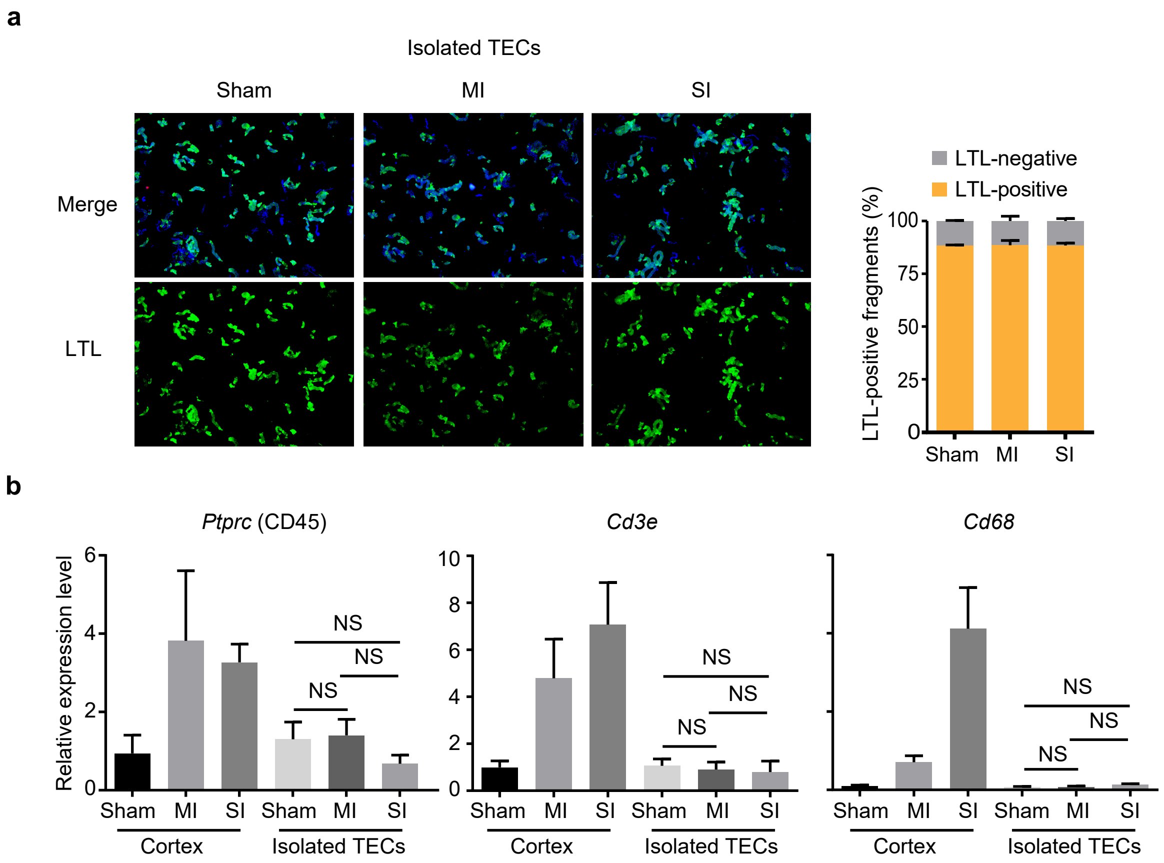

Supplementary Figure 3. Purification of isolated TECs.

a Immunofluorescence staining of LTL-positive tubules (left). Quantitative analysis of LTL-positive (right). **b** qRT-PCR analysis of *Ptprc* (CD45), *Cd3e*, and *Cd68* expression in isolated TECs. Data are represented as means ± SEM. Statistically significant differences were determined by two-tailed unpaired Student's *t*-test. From left to right for *Ptprc* : NS $P = 0.2151$, NS $P = 0.8773$, NS $P = 0.1589$. From left to right for *Cd3e* : NS $P = 0.6729$, NS $P = 0.7351$, NS$P = 0.8573$. From left to right for *Cd68* : NS $P = 0.0947$, NS $P = 0.8415$, NS $P = 0.1090$. NS: not significant. $n = 5$ biologically independent samples. Source data are provided as a Source Data file.

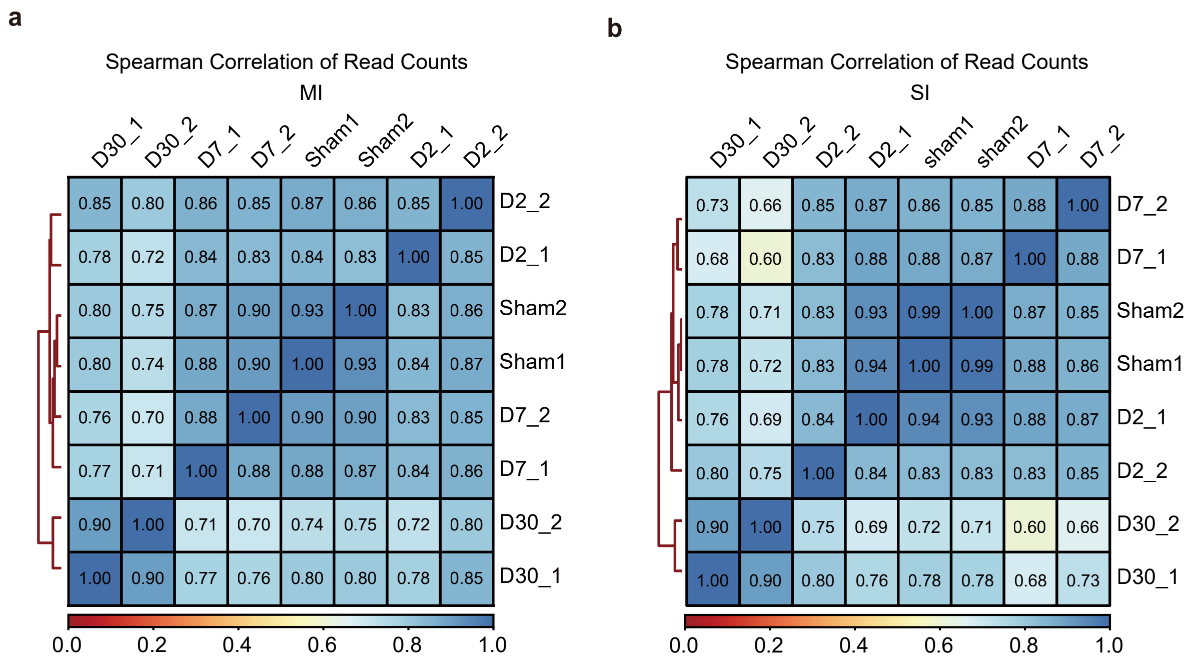

Supplementary Figure 4. a, **b** Heatmaps showing Spearman's correlation coefficients between biological replicates in mild injury and severe injury. The color intensity indicates the strength of correlation.

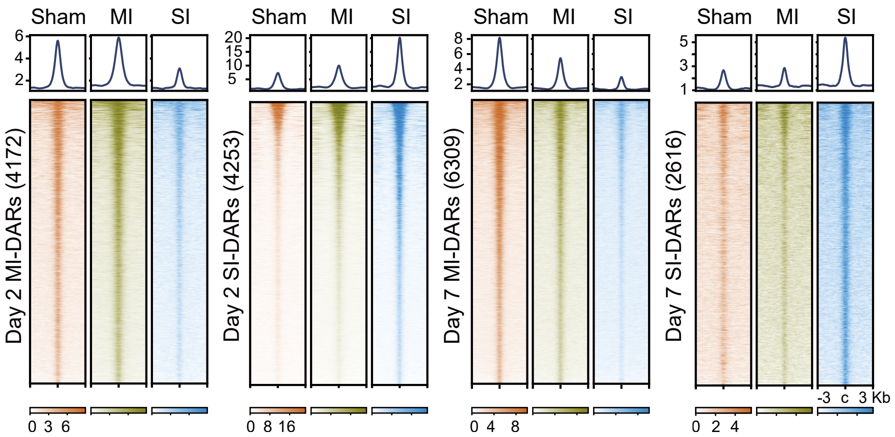

Supplementary Figure 5. Visualization of ATAC-seq signals in Sham, MI, and SI TECs centered on peak summits ± 3 Kb. Peaks are ordered vertically by ATAC-seq signal strength. The signal strength is denoted by color intensities.

Supplementary Table 1. Characteristics of AKI patients.

Patient No.	Etiology	Biopsy BUN (mmol/L)	Biopsy Scr (μmol/L)	Peak Scr (μmol/L)	AKI or AKD	Peak stage	ATI severity
1	1	11	182	213	2	1	mild
2	4	9.7	167	178	1	1	mild
3	1	16.09	486.2	486.2	1	3	severe
4	1	17.83	541	541	1	3	severe
5	4	14.84	379	426.5	1	3	severe
6	2	18.1	387	507.3	1	3	severe
7	1 and 2	22.62	988.9	988.9	1	3	severe
8	2	4.3	376	852	1	3	severe
9	4	15.89	287	580	1	3	severe
10	1	6.94	120.1	126	1	1	mild
11	3	11.98	246.8	282.93	1	2	mild
12	2	5	168.8	168.8	2	1	mild
13	2	3.4	75.5	84	3	/	mild
14	2	6.1	113	122.6	2	1	mild
15	4	3.95	51.7	69	2	1	mild
16	2	9.3	166.3	683.6	2	3	mild
17	2	6.06	177	365	1	3	mild
18	1	6.1	464	1702	1	3	mild
19	2	8	133	224	1	3	mild
20	3	12.7	349.07	913	1	3	severe
21	2	9.4	259	464	2	3	severe
22	2	9.3	222.3	290	2	1	severe
23	1 and 2	5.1	96.8	1241	1	3	severe
24	3	10.8	400	1415	1	3	severe
25	1 and 2	4.72	262	386	1	3	severe
26	4	19.49	224.4	995.9	1	3	severe
27	1	7.7	189.2	685	1	3	severe
28	1	9.94	722.5	1345.9	1	3	severe

Notes:

Etiology: 1/ischemia; 2/nephrotoxic; 3/others; 4/unknown

AKI/AKD: AKI = 1; AKD = 2; Non_AKI or Non_AKD = 3

Supplementary Table 2. Primers for RT-PCR

Gene name	Forward	Reverse
Mus. *Fn1*	ACAAGGTTCGGGAAGAGGTT	CCGTGTAAGGGTCAAAGCAT
Mus. *Col3a1*	ACAGCTGGTGAACCTGGAAG	ACCAGGAGATCCATCTCGAC
Mus. *Lnc2*	GACTTCCGGAGCGATCAGTT	CTGATCCAGTAGCGACAGCC
Mus. *Havcr1*	TCCACACATGTACCAACATCAA	GTCACAGTGCCATTCCAGTC
Mus. *Actb*	TTGCTGACAGGATGCAGAAG	ATCCACATCTGCTGGAAGGT
Mus. *Rxra*	GTCGCAGACATGGACACCAA	CGTTGGAGAGTTGAGGGACG
Mus. *Ptprc*	TGGCCTTTGGATTTGCCCTT	CTGTTGTGCTCAGTTCATCACT
Mus. *Cd68*	GCCTAGTCCAAGGTCCAAGG	ACAGATATGCCCCAAGCCTTT
Mus. *Dd3e*	GTGGAACACTTTCTGGGGCA	TCAATGTTCTCGGCATCGTCC
