# Supplementary material for: Chromatin accessibility dynamics dictate renal tubular epithelial cell response to injury
Source: Nat Commun. 2022 Nov 28;13:7322. doi: 10.1038/s41467-022-34854-w (PMC9705299; doi:10.1038/s41467-022-34854-w)
Supplement: Supplementary file 2 — Reporting Summary [file 41467_2022_34854_MOESM2_ESM.pdf]

nature portfolio

Corresponding author(s): Yupeng Chen; Li Yang; Lirong Zhang

Last updated by author(s): Sep 22, 2022

Reporting Summary

Nature Portfolio wishes to improve the reproducibility of the work that we publish. This form provides structure for consistency and transparency in reporting. For further information on Nature Portfolio policies, see our Editorial Policies and the Editorial Policy Checklist.

Statistics

For all statistical analyses, confirm that the following items are present in the figure legend, table legend, main text, or Methods section.

n/a	Confirmed	
☐	☒	The exact sample size (*n*) for each experimental group/condition, given as a discrete number and unit of measurement
☐	☒	A statement on whether measurements were taken from distinct samples or whether the same sample was measured repeatedly
☐	☒	The statistical test(s) used AND whether they are one- or two-sided<br>*Only common tests should be described solely by name; describe more complex techniques in the Methods section.*
☒	☐	A description of all covariates tested
☐	☒	A description of any assumptions or corrections, such as tests of normality and adjustment for multiple comparisons
☐	☒	A full description of the statistical parameters including central tendency (e.g. means) or other basic estimates (e.g. regression coefficient) AND variation (e.g. standard deviation) or associated estimates of uncertainty (e.g. confidence intervals)
☐	☒	For null hypothesis testing, the test statistic (e.g. *F*, *t*, *r*) with confidence intervals, effect sizes, degrees of freedom and *P* value noted<br>*Give P values as exact values whenever suitable.*
☒	☐	For Bayesian analysis, information on the choice of priors and Markov chain Monte Carlo settings
☐	☒	For hierarchical and complex designs, identification of the appropriate level for tests and full reporting of outcomes
☐	☒	Estimates of effect sizes (e.g. Cohen's *d*, Pearson's *r*), indicating how they were calculated

Our web collection on statistics for biologists contains articles on many of the points above.

Software and code

Policy information about availability of computer code

Data collection	For ATAC-seq, ChIP-seq, and RNA-seq data, FASTQ files were trimmed with trimGalore (v1.18).
Data analysis	For ATAC-seq and ChIP-seq data, filtered reads were aligned to mm10 with Bowtie2 (v2.3.5.1). Peak calling was performed using MACS2 (v2.2.7.1). Differential peaks between tissue pairs were identified using HOMER(v4.10) with P value < 0.01 as cutoff. HOMER was also used for motif enrichment analysis. For ATAC-seq only, correlation analysis was performed using deepTools (v3.4.3) and reads mapped to mitochondrial DNA were removed by removeChrom. For dynamic ATAC peak identification among different stages, we conducted a soft clustering analysis with the Mfuzz R package. Chromatin accessibility data showing a pertinence membership > 51% at a given stage were grouped into four Mfuzz clusters. For RNA-seq, adaptor-trimmed RNA-seq reads were aligned to the mouse reference genome (GRCm38/mm10) with HISAT2 (v2.1.0). Mapped reads were quantified using featureCounts (v1.6.0). Differential expression was calculated with the DESeq2 package. Differentially expressed genes (DEGs) were determined using a cutoff of > 1.5-fold change with adjusted P < 0.05. General statistical data was analyzed using GraphPad Prism 8.0 software. The degree of interstitial fibrosis was assessed with MTS-stained kidney sections using Image-Pro Plus 6.0 software.

For manuscripts utilizing custom algorithms or software that are central to the research but not yet described in published literature, software must be made available to editors and reviewers. We strongly encourage code deposition in a community repository (e.g. GitHub). See the Nature Portfolio guidelines for submitting code & software for further information.

Data

Policy information about availability of data

All manuscripts must include a data availability statement. This statement should provide the following information, where applicable:

- Accession codes, unique identifiers, or web links for publicly available datasets
- A description of any restrictions on data availability
- For clinical datasets or third party data, please ensure that the statement adheres to our policy

The main data supporting the findings of this study are available within this Article, it's Supplementary Information and Source Data. ATAC-seq, ChIP-seq, and RNA-seq raw data files are available in the Gene Expression Omnibus (GEO) with the accession number GSE197815 (RNA-Seq: GSE197812, ATAC-Seq: GSE197814, H3K27ac ChIP-Seq: GSE213526, RXRα ChIP-Seq: GSE197813). ATAC-Seq, ChIP-seq, and RNA-seq reads were aligned to the mouse reference genome (GRCm38/mm10). All other data can be found within the main manuscript or the source data file. Source data are provided with this paper.

Human research participants

Policy information about studies involving human research participants and Sex and Gender in Research.

Reporting on sex and gender	Twenty-eight patients diagnosed with renal tubular acute injury (ATI) or acute tubular necrosis (ATN) were recruited in the study. There were 12 patients in the mild injury group and 16 patients in the severe injury group. Information of sex and gender are summarized in Supplementary Table 1.
Population characteristics	AKI was defined according to the Kidney Disease Improving Global Outcomes (KDIGO) criteria (https://kdigo.org/guidelines/acute-kidney-injury/). AKI stages were defined using the KDIGO AKI stage Scr definitions. AKD and its stages was defined by the Acute Disease Quality Initiative (ADQI) consensus as AKI stage 1 or greater (as defined by KDIGO) that is present between 7 and 90 days after an AKI episode. Clinical characteristics and pathological evaluations of the enrolled patients are summarized in Supplementary Table 1.
Recruitment	Patients in the Peking University First Hospital from 2007 to 2020 who were diagnosed with renal tubular acute injury (ATI) or acute tubular necrosis (ATN) were recruited in the study. The degree of renal tubular acute injury was assessed by two renal pathologists in a blinded manner. A 0 to 4+ scale scoring system was used based on the percentage of renal tubules affected by the loss of tubule epithelial cell brush border and tubular necrosis and/or apoptosis (0 = no lesion, 1+ = ≤ 25%, 2+ = > 25 to 50%, 3+ = > 50 to 75%, 4+ = > 75 to < 100%)[PMID: 32967729]. Scores of 1 and 2 were defined as mild ATI, and scores of 3 and 4 as severe ATI.
Ethics oversight	The study design and conduct in accordance with Chinese law and the criteria set by the Declaration of Helsinki. The protocol concerning the use of patient samples in this study was approved by the Biomedical Research Ethics Committee of Peking University First Hospital (approval number: 2017[1280]), and informed consent was obtained from all participants.

Note that full information on the approval of the study protocol must also be provided in the manuscript.

Field-specific reporting

Please select the one below that is the best fit for your research. If you are not sure, read the appropriate sections before making your selection.

☒ Life sciences ☐ Behavioural & social sciences ☐ Ecological, evolutionary & environmental sciences

For a reference copy of the document with all sections, see nature.com/documents/nr-reporting-summary-flat.pdf

Life sciences study design

All studies must disclose on these points even when the disclosure is negative.

Sample size	For the animal study, eight-week-old C57BL/6 male mice were used in this study. Sample size choice was based on previous studies (PMID: 30325740, PMID: 32636391, PMID: 20436483, and PMID: 32404507), not predetermined by a statistical method. For animal studies (Fig 5,6), five (or more) mice per treatment group were used in independent experiments. For biological studies, three to five samples for each group were used in kidney tissues relatived experiments: qPCR and RXRα expression determination. Sample sizes were indicated in the figure, figure legend of each Figure and Supplementary Figure. All of experiment results detected a statistically significant difference P<0.05.
Data exclusions	No data were excluded.
Replication	We confirm all attempts at replication were successful. For each assessment/measurement, similar results were consistently obtained in more than 3 independent experiments with more than 3 biological replicates, and all experiments are reported in the manuscript.
Randomization	All samples were randomly allocated into experimental groups.

Blinding | All the investigators were blinded to group allocation during data collection and analysis.

Reporting for specific materials, systems and methods

We require information from authors about some types of materials, experimental systems and methods used in many studies. Here, indicate whether each material, system or method listed is relevant to your study. If you are not sure if a list item applies to your research, read the appropriate section before selecting a response.

Materials & experimental systems

n/a	Involved in the study
☐	☒ Antibodies
☒	☐ Eukaryotic cell lines
☒	☐ Palaeontology and archaeology
☐	☒ Animals and other organisms
☒	☐ Clinical data
☒	☐ Dual use research of concern

Methods

n/a	Involved in the study
☐	☒ ChIP-seq
☒	☐ Flow cytometry
☒	☐ MRI-based neuroimaging

Antibodies

Antibodies used

Primary antibodies against RXRα (ET7108-99, Huabio), α-SMA (ab5694, Abcam), Collagne-1(ab34710, Abcam), and Fibronectin (F3648, Sigma) were used for immunohistochemistry;
Primary antibodies against KIM-1 (AF1817, R&D System) was used for immunofluorescence;
Primary antibodies against RXRα (ET7108-99, Huabio) and H3K27ac(ab4729, Abcam) were used for ChIP;
Alexa Fluor 555-conjugated secondary antibodies (Invitrogen, 2273776, 1:1000 dilution) were used for immunofluorescence.

Validation

The antibodies were chosen based on the experience in our own laboratory and/or the provider's catalogy information on the ones used in previously published studies in the literature.

Antibodies against RXRα (ET7108-99, Huabio) was tested in RXRA-knockout cell lines in our own laboratory . (IHC: dilution 1:200; ChIP-seq: 2 μg)
Species Reactivity: Human, Rat, Mouse
Suitable for: Human, Rat, Mouse
https://huabioresearch.com/datasheet/ET7108-99

Antibodies against KIM-1 (AF1817, R&D System) was validated in multiple studies. (IF: dilution 1:200)
Selected publications:
PMID: 31710314; PMID: 33951465; PMID: 35986026

Antibodies against α-SMA was validated in multiple studies. (IHC: dilution 1:100)
Selected publications:
PMID: 33523909; PMID: 33537098; PMID: 33567287.

Antibodies against Collagne-1 was validated in multiple studies. (IHC: dilution 1:800)
Selected publications:
PMID: 32302523; PMID: 33972536; PMID: 33397952; PMID: 33863879

Antibodies against Fibronectin was validated in multiple studies. (IHC: dilution 1:1000)
Selected publications:
PMID: 35973996; PMID: 35931031; PMID:34099830

Antibodies against H3K27ac (Abcam cat# ab4729, lot# GR3252404-1, 2 μg for ChIPseq)
https://www.abcam.com/histone-h3-acetyl-k27-antibody-chip-grade-ab4729.html

Animals and other research organisms

Policy information about studies involving animals; ARRIVE guidelines recommended for reporting animal research, and Sex and Gender in Research

Laboratory animals

Mice were housed on a 12h light-dark cycle at 21–25°C with 30–70% humidity and allowed free access to food and water except as noted. Mice were randomly assigned to experimental groups.

Wild animals	No wild animals were used.
Reporting on sex	Eight-week-old C57BL/6 male mice were used in this study. Due to the substantial difference in susceptibility to IRI injury between. male and female mice (PMID: 22993069), male mice were exclusively used to reduce total numbers of mice required for statistical analysis.
Field-collected samples	This study did not involve samples collected from the field.
Ethics oversight	All mouse experiments were approved by the Ethical Committee of Tianjin Medical University.

Note that full information on the approval of the study protocol must also be provided in the manuscript.

ChIP-seq

Data deposition

☒ Confirm that both raw and final processed data have been deposited in a public database such as GEO.

☒ Confirm that you have deposited or provided access to graph files (e.g. BED files) for the called peaks.

Data access links<br>*May remain private before publication.*	Sequencing data were deposited in the Gene Expression Omnibus (GSE197815) and are available on the web (https://www.ncbi.nlm.nih.gov/geo/query/acc.cgi?acc=GSE197815).
Files in database submission	ChIP-seq Sham and IRI (mild injury and severe injury): RXRα and H3K27ac
Genome browser session<br>(e.g. UCSC)	All raw (fastq) and processed data (bigwig) are available in GEO.

Methodology

Replicates	We performed 1 replicate in this assay.
Sequencing depth	ChIP-seq: pair-end 150 bp<br><br>RXRα ChIP-seq raw read counts:<br>Unique reads number of Sham input is 20530424 and Sham RXRα ChIP 20433332.<br>Unique reads number of MI input is 21920656 and MI RXRα ChIP 30439620.<br>Unique reads number SI input is 20956856 and SI RXRα ChIP 33057814.<br>Unique reads number of SI_Bex input is 22192568 and SI RXRα ChIP is 31271040.<br><br>H3K27ac ChIP-seq raw read counts:<br>Unique reads number of day2 MI input is 28142980 and H3K27ac ChIP is 10279310.<br>Unique reads number of day7 MI input is 25986328 and H3K27ac ChIP is 12024520.<br>Unique reads number of day2 SI input is 20981554 and H3K27ac ChIP is 16122150.<br>Unique reads number of day7 SI input is 16249484 and H3K27ac ChIP is 14591646.
Antibodies	We used H3K27ac antibody (ab4729, Abcam, 2μg) for ChIP-seq assay.<br>We used RXRα antibody (ET7108-99, Huabio, 2μg) for ChIP-seq assay.
Peak calling parameters	macs2 callpeak --keep-dup all -t $i.bam -g mm -f BAMPE --outdir macs2/ -n $i -m 2 500
Data quality	ChIP-seq quality control was performed by FastQC (v0.11.9) software.
Software	Trimmed clean reads were aligned to the mouse reference genome (mm10) with Bowtie (v2.3.5.1). MACS2 (v2.2.7.1) was used for ChIP-seq peak calling and BED file generation. BigWig files were derived from deepTools (v3.4.3). Differential accessible regions were analyzed on normalized trimmed counts using HOMER with P value < 0.01 as cutoff. Annotation of peaks was performed using HOMER.
